# Prediction of conical collimator collision for stereotactic radiosurgery

**DOI:** 10.1002/acm2.12963

**Published:** 2020-07-06

**Authors:** Jeonghoon Park, Ryan McDermott, Sangroh Kim, M. Saiful Huq

**Affiliations:** ^1^ Department of Radiation Oncology University of Pittsburgh School of Medicine and UPMC Hillman Cancer Center Pittsburgh PA USA; ^2^ Department of Radiation Oncology The Medical Center at Bowling Green Bowling Green KY USA; ^3^ Department of Radiation Oncology Virginia Mason Medical Center Seattle WA USA

**Keywords:** Beam's eye view, collision prediction, conical collimator, stereotactic radiosurgery

## Abstract

The purpose of this study is to predict the collision clearance distance of stereotactic cones with treatment setup devices in cone‐based stereotactic radiosurgery (SRS). The BrainLAB radiosurgery system with a Frameless Radiosurgery Positioning Array and dedicated couch top was targeted in this study. The positioning array and couch top were scanned with CT simulators, and their outer contours of were detected. The minimum clearance distance was estimated by calculating the Euclidian distances between the surface of the SRS cones and the nearest surface of the outer contours. The coordinate transformation of the outer contour was performed by incorporating the Beam's Eye View at a planned arc range and couch angle. From the minimum clearance distance, the collision‐free gantry ranges for each couch angle were sequentially determined. An in‐house software was developed to calculate the clearance distance between the cone surface and the outer contours, and thus determine the occurrence of a collision. The software was extensively tested for various combinations of couch and arc angles at multiple isocenter locations for two combinations of cone‐couch systems. A total of 50 arcs were used to validate the calculation accuracies of the software for each system. The calculated minimum distances and collision‐free angles from the software were verified by physical measurements. The calculated minimum distances were found to agree with the measurements to within 0.3 ± 0.9 mm. The collision‐free arc angles from the software also agreed with the measurements to within 1.1 ± 1.1° with a 5‐mm safety margin for 20 arcs. In conclusion, the in‐house software was able to calculate the minimum clearance distance with <1.0 mm accuracy and to determine the collision‐free arc range for the cone‐based BrainLab SRS system.

## 1 Introduction

Stereotactic Radiosurgery (SRS) using a conical collimator is an effective radiation treatment method for small intracranial targets, which allows the delivery of high radiation dose to the lesion with submillimeter accuracy and sharp dose fall‐off. The use of stereotactic cones began in the 1980s in favor of (a) tertiary collimation system to provide small field sizes, (b) superior lateral penumbra compared to rectangular collimators, (c) spherical isodose distributions conformal to the tumor shape.^1^ Although multileaf‐collimator (MLC)‐based SRS became prominent recently, cone‐based SRS is still being used to treat small intracranial targets such as small benign or metastatic tumors, or functional diseases like trigeminal neuralgia.^2^, ^3^, ^4^

The most advantageous characteristic of cone‐based SRS system is a sharp dose fall‐off by minimizing lateral scattering owing to its physically long and narrow design.^5^, ^6^, ^7^ However, due to the protruding cone design, the gantry clearance space is quite smaller than that of a general MLC‐based treatment system. Consequently, there are higher chances of collision between the cone and the patient and/or setup devices in cone‐based treatments. However, it is quite challenging to find the collision‐free gantry/couch angle combination manually due to the nature of complex three‐dimensional (3D) geometries of patients with gantry and couch motions in the 3D coordinate system.

There were many previous studies on the prediction of collision among gantry, couch and patients in various systems to avoid any harm to the patient and prevent replanning. Humm et al. proposed an analytic approach and developed a software to detect the collision of gantry/couch and gantry/patient by modeling a couch surface and patient geometry mathematically.^8^, ^9^ Hua et al. also developed a mathematical collision prediction model for BrainLAB micro MLC and BRW SRS system by approximating the micro MLC rotation space as a circle and the couch as a rectangle.^10^ Nioutsikou et al presented an analytic solution to the Precise (Elekta AB, Stockholm, Sweden) LINAC and Pinnacle (Philips Medical Systems, Eindhoven, Nederland) treatment planning system (TPS) using a two‐dimensional (2D) collision map.^11^ Tsiakalos et al developed an OpenGL‐based room's eye view simulation solution to detect a collision by graphical modeling of the LINAC.^12^ Becker et al created an allowable combination of gantry and couch angles on a polar graph, and verified with vertical/lateral couch shifts for Varian, Siemens and Elekta LINAC.^13^, ^14^ Padilla et al evaluated the clearance of the patient and immobilization device against the gantry using surface reconstruction with Kinect^TM^ (Microsoft, Redmond, WA) camera. Cardan et al also demonstrated similar collision avoidance framework using three Kinect cameras.^15^, ^16^ Yu et al developed the full CAD model of TrueBeam (Varian Medical Systems, Palo Alto, CA) LINAC and 3D scanned patient surface and verified the automation of non‐coplanar beam geometries, which has been commercialized as HyperArc.^17^ Recently, Mann et al also developed a CAD‐based solution for the Edge (Varian Medical Systems, Palo Alto, CA) LINAC with an extension to the ESAPI (Eclipse Scripting Application Programming Interface)‐based automation.^18^ Similarly, a virtual simulator was developed for the detection of collision among the proton beam nozzle, patient, and couch.^19^

While most of the existing collision detection method requires 3D modeling, CAD technique, or an external camera, this study introduced the mathematical operation of the contours instead of such complex techniques for the practical implementation in a clinic. The necessity of collision prevention is more emphasized for the cone‐based SRS since it introduces a protruding conical collimator which consequently leads to tighter gantry clearance margin. Multileaf‐collimator‐based treatment has relatively less chance of interference because there is more clearance to the gantry head. This study is aimed at developing a robust collision detection algorithm and software for cone‐based SRS, which can assist the treatment planner to test any gantry and couch angle combination and to find collision‐free arc range at the time of treatment planning.

## 2 Materials and methods

### 2.A Characteristics of frameless SRS system

**Fig. 1:**
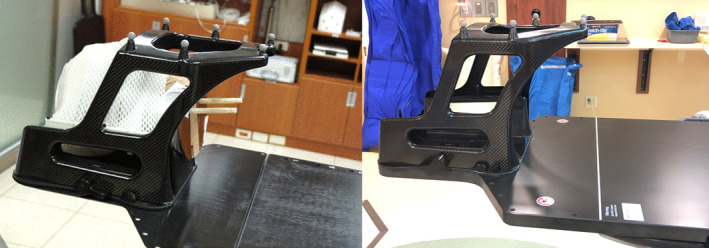
Brainlab frameless SRS system (Left) on the Brainlab imaging couch top frameless extension, (Right) on the Universal Couch Extension attached to the Varian PerfectPitch Couch.

This study utilized the BrainLAB frameless SRS system. It consists of the Imaging Couch Top (ICT) Frameless Extension for BrainLAB ExacTrac^TM^ system or the Frameless SRS Universal Couch Extension for Varian IGRT couch system, the Frameless SRS mask base, the Frameless Radiosurgery Positioning Array (FRPA), and stereotactic localizer (Fig. 1). The ICT Frameless Extension attaches on the BrainLAB ExacTrac Robotic Couch frame and provides a support in the cranial treatment. The Frameless SRS Universal Couch Extension attaches on the Varian IGRT couch top and functions the same as the ICT extension. The Frameless SRS Mask Base is fixed above both couch extensions to provide patient fixation using a thermoplastic mask. FRPA is used for initial patient alignment and in‐treatment monitoring using six infrared (IR) markers. The stereotactic localizer is an N‐shaped localization box used for CT/MR imaging. It realigns CT/MR images to the BrainLAB stereotactic coordinate system from the DICOM coordinate system using three pairs of fiducial markers. Coordinate localization is available using a localization function in the iPlan TPS or an image registration with the couch template of ExacTrac and BrainLAB Elements TPS. While the DICOM coordinate has a variable origin set in the CT simulation, the localized image set has a universal origin regardless of CT origin or couch top. The same concept applies to the treatment coordinate.

### 2.B Modeling of SRS Frame Structure

The BrainLAB Frameless SRS system with ExacTrac Robotic couch at Baylor Scott and White Medical Center (BSW) and the same system with Varian PerfectPitch^TM^ Couch (PPC) with 6 degrees of freedom (DoF) at the University of Pittsburgh Medical Center (UPMC) Hillman Cancer Center were used in this study. Two separate CT scans were performed for each system using the GE Optima CT580 at BSW and the GE Discovery RT CT scanner at UPMC Hillman Cancer Center with a 1.25‐mm slice thickness and 65‐cm field‐of‐view (FOV). First, the SRS mask base and ICT Extension or Universal Couch Extension with a stereotactic CT localizer was scanned. The images were sent to the iPlan TPS to define a BrainLAB coordinate, and they were exported to Eclipse v15.6 (Varian Medical Systems, Palo Alto, CA) TPS. Then, second image sets were acquired for the same configuration with FRPA instead of a localizer and sent to Eclipse directly. Image registration was performed between two image sets with a region of interest (ROI) around the SRS mask base and couch extensions.

**Fig. 2:**
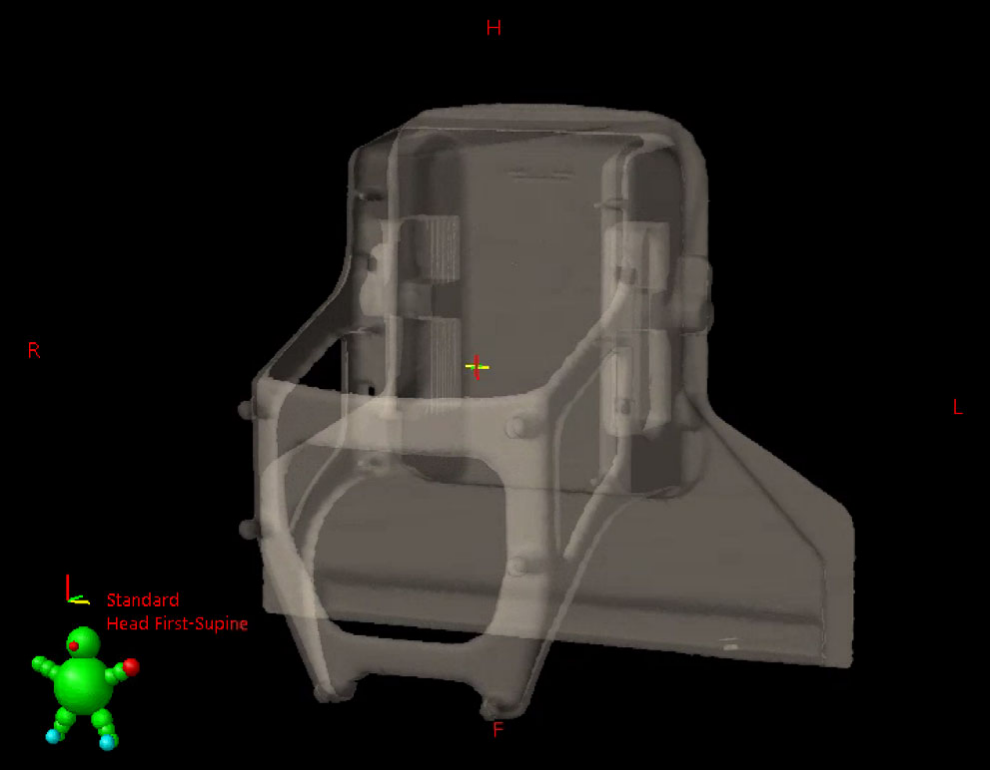
Delineation of the outer contour of imaging couch top couch extension and frameless radiosurgery positioning array in Eclipse.

In the second CT image, the outer contour of the couch extension and FRPA were contoured in Eclipse using the “Search Body” function with a −650 HU threshold to delineate collisional objects. The outer contour was smoothed and manually corrected. Then, it was transferred to the first CT image through the image registration so that the outer contour is represented in the Brainlab coordinate. It was applied to both BrainLAB ICT and Varian PPC extensions. This procedure was required to render the FRPA and couch contours in the Brainlab coordinate since the CT localizer and the FRPA cannot be used simultaneously in the imaging. The final structure set with the surface contour of FRPA and both couch extensions was exported in DICOM format (Fig. 2).

### 2.C Beam's eye view transformation

**Fig. 3:**
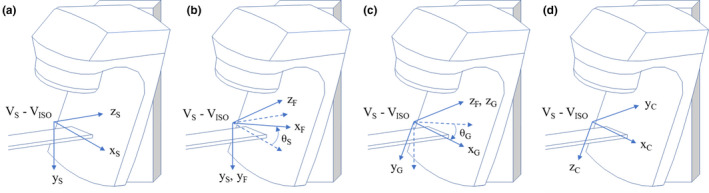
Coordinate transformation from the patient coordinate system to the collimator coordinate system. (a) Translation to the isocenter in the patient support coordinate system, (b) Rotation of the patient support coordinate system around y axis by −θ_S_ back to the machine fixed coordinate, (c) Rotation of the machine fixed coordinate system by θ_G_ to the gantry coordinate system, (d) Exchange of coordinate convention to the collimator coordinate system.

Beam's eye view (BEV) was employed to define the search area of the minimum distance between the SRS position array surface and the cone surface for an arbitrary gantry‐couch combination. Beam's eye view is a very useful tool to visualize the patient anatomy and to design the beam aperture in the computerized treatment planning.^20^, ^21^, ^22^ Most of the BEV application is limited to the 2D plane, that is, collimator X and Y axis, but depth information along the collimator Z axis can provide useful information on the distance from the target to an object. In general, BEV of an object is created through the successive coordinate transformations from the patient coordinate system to the collimator coordinate system as shown in Fig. 3 and Eq. (1):^23^

(1) $${\left[V\right]}_{C}=R_{Z}\left(\theta_{C}\right)\cdot R_{Z}\left(\theta_{G}\right)\cdot R_{Y}\left(-\theta_{S}\right)\cdot \left({\left[V\right]}_{S}-{\left[\text{IC}\right]}_{S}\right)$$

where [V]_C_ is a new coordinate in the collimator coordinate, [V]_S_ and [IC]_S_ are the coordinates of the arbitrary point to be transformed and the isocenter in the patient support coordinate system, and R is the rotational matrix about the axis, θ_S_, θ_G,_ and θ_C_ are the patient support angle, gantry angle, and collimator angle, respectively. The perspective transformation along the z‐axis of the collimator coordinate was not applied here because it warps x, y coordinates used to estimate the Euclidean distance. Note that the perspective transformation is only relevant for more realistic BEV visualization. Detailed description of the coordinate transformation follows in Eq. (2). Collimator rotation was set to zero degree due to the symmetry of the cones. The Varian IEC convention is used for the BEV coordinate system in Eqs. (1) and (2).

(2) $$\left[\begin{array}{c} V_{X,C}\\ V_{Y,C}\\ V_{Z,C} \end{array}\right]=\left[\begin{array}{ccc} 1 & 0 & 0\\ 0 & 0 & 1\\ 0 & -1 & 0 \end{array}\right]\left[\begin{array}{ccc} \cos\left(\theta_{G}\right) & \sin\left(\theta_{G}\right) & 0\\ -\sin\left(\theta_{G}\right) & \cos\left(\theta_{G}\right) & 0\\ 0 & 0 & 1 \end{array}\right]\left[\begin{array}{ccc} \cos\left(\theta_{S}\right) & 0 & \sin\left(\theta_{S}\right)\\ 0 & 1 & 0\\ -\sin\left(\theta_{S}\right) & 0 & \cos\left(\theta_{S}\right) \end{array}\right]\left(\left[\begin{array}{c} V_{X,S}\\ V_{Y,S}\\ V_{Z,S} \end{array}\right]-\left[\begin{array}{c} IC_{X,S}\\ IC_{Y,S}\\ IC_{Z,S} \end{array}\right]\right)$$

### 2.D Determination of clearance distance and collision

**Fig. 4:**
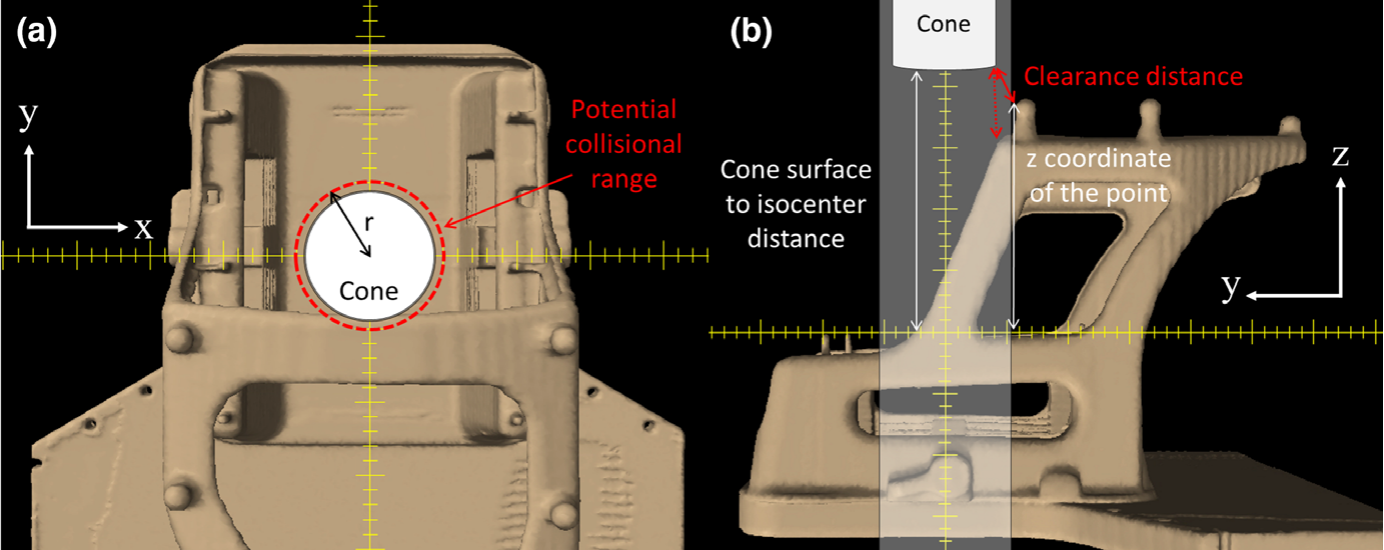
Determination of a clearance distance from the Beam's eye view coordinate of the outer contour (a) Arbitrary point within the cone radius + detection margin will be of potential collision, (b) Clearance distance is the short distance between the cone and the points found in (a) shown as shaded volume.

BrainLAB SRS cones at UPMC Hillman Cancer Center and Varian Integrated Conical Collimator Verification & Interlock (ICVI) cones at BSW were used in this study. The diameters of the cones were measured as 67.5 and 74.0 mm, respectively. Perpendicular distances between the cone surface and isocenter were measured as 26.6 cm for BrainLAB cones in TrueBeam STx^TM^ and 25.6 cm for Varian ICVI cones in Novalis Tx^TM^, respectively. Clearance distance of the cone at the specific couch and gantry angle was determined from the Euclidean distance between the cone surface or edge and the nearest outer contour. First, the radial distance from the isocenter to the arbitrary point of the outer contour was calculated using x, y coordinates in the BEV. If the distance is shorter than the radius of the cone plus a detection margin (5 mm by default), that is, the point lies within the projection of the cone plus margin, the point will be selected as a potential collision point [Fig. 4(a)]. Then, the clearance distance is obtained as a minimum Euclidean distance from the cone surface or edge to arbitrary points found in the previous step [Fig. 4(b)]. If the z coordinate of the point in the BEV is larger than the distance between the cone surface and the isocenter, a collision will occur at that position. Since the outer contour usually consists of tens of thousands of points, this operation has to be repeated for every point and a minimum distance will be taken as a clearance distance.

**Fig. 5:**
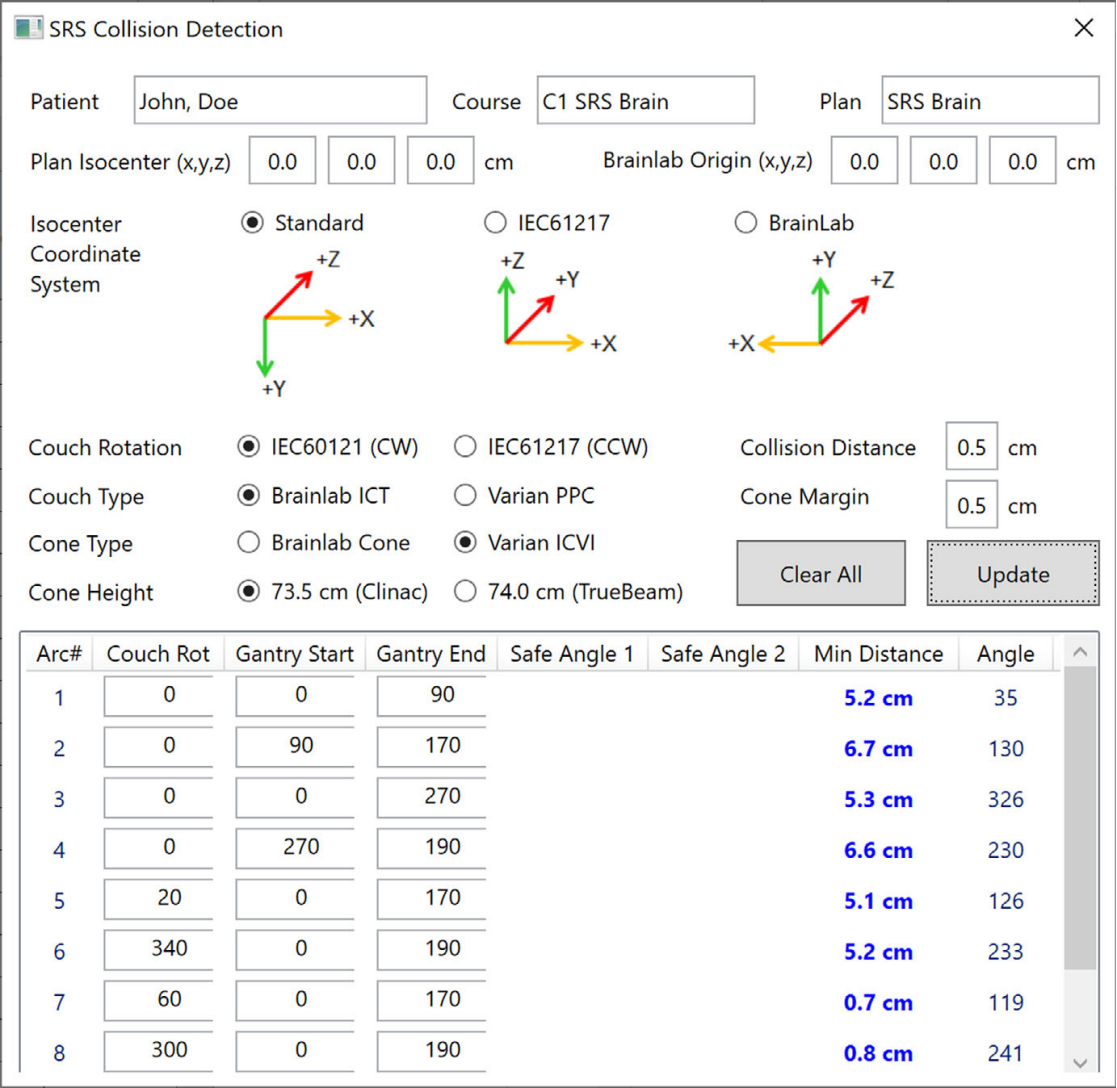
In‐house software was developed to calculate a clearance distance between the cone and the outer contour. Clearance distances and relevant angles are calculated at every 1° interval. Various combinations of coordinate system, couch type, cone type, and machine type are supported.

We developed an in‐house software using Microsoft Visual C#.Net 2013 to convert the outer contour to the BEV coordinates and to calculate the clearance distance (Fig. 5). Evil DICOM C# library was used to open an RT structure DICOM file and parse contour points.^24^ The software utilizes the preprocessed FRPA outer contour combined with the Brainlab and Varian couch contour. Arbitrary combination with Brainlab cone or Varian ICVI cone is available for the Varian Clinac or TrueBeam machine. In the software, the clearance distances are evaluated at every 1‐degree interval from the start angle to the end angle of the arc. It provides the minimum cone distances and the relevant gantry angle. In case collision occurs, the collision‐free arc range is calculated with a predefined safety margin. It also supports BrainLAB stereotactic images that were not localized in iPlan by translating the outer contours to BrainLAB coordinates, with the origin known from the image registration with a couch extension template. Six‐dimensional (6D) corrections during the patient setup such as couch translation, roll, pitch, and yaw are not considered in the determination of collision.

### 2.E Verification of the proposed method

The proposed method was verified by measuring physical distances between the cone and the FRPA or couch at an arbitrary isocenter with multiple couch angle, arc range combinations. As aforementioned, two test systems were employed in this study – (a) BrainLAB ExacTrac couch system with ICT Frameless Extension using Varian ICVI cone in BSW and (b) Varian PPC system with Universal Couch Extension using BrainLAB cone in Hillman Cancer Center. FRPA was attached on the couch extension for both cases. Test plans in Table 1 were made for each system using the Eclipse v13.6 in BSW and v15.6 in UPMC Hillman Cancer Center; total of 100 arcs, or 50 arcs for each system were verified. These plans were imported in the Brainlab ExacTrac (v 6.6.0) system. Initial couch positions of each isocenter were aligned using an automatic positioning function of ExacTrac software by detecting IR markers of FRPA. Physical measurement of the clearance distance was performed with a ruler at the minimum clearance gantry angle predicted by the software. The minimum clearance angle and distance were updated if a shorter distance was found. In the meantime, the accuracy of collision‐free arc range was also verified for 20 arcs equivalent to 40 potential collisional gantry angles at five isocenters with a 5 mm safety margin using the Varian PPC system (Table 2). These arcs are designed to collide with a couch top or FRPA in the middle of the arc. Actual gantry angles at which the distances between the cone and a couch top or FRPA become 5 mm first and last were recorded in the measurement using a custom gauge.

**Table 1:** Isocenter, couch angle, and arc ranges of test treatment plans used for the verification of clearance distance. Eclipse isocenter coordinate is based on Varian IEC convention.

Plan no.	Eclipse isocenter (cm)			Couch angle (°)	Gantry start (°)	Gantry end (°)
	X (+: Lt)	Y (+: Post)	Z (+: Sup)			
1	0.00	0.00	0.00	0	0	90
				0	90	170
				0	0	270
				0	270	190
				20	0	170
				340	0	190
				60	0	170
				300	0	190
				90	0	170
2	0.00	−1.00	2.00	0	0	170
				0	0	190
				20	0	170
				340	0	190
				55	0	170
				305	0	190
				90	0	170
3	0.00	−2.00	4.00	0	0	170
				0	0	190
				20	0	170
				340	0	190
				50	0	170
				310	0	190
				90	0	170
4	3.00	1.00	3.00	0	0	170
				0	0	190
				20	0	170
				340	0	190
				60	0	170
				315	0	190
				90	0	170
5	5.00	−1.00	3.00	0	0	170
				0	0	190
				20	0	170
				340	0	190
				65	0	170
				90	0	170
6	−3.00	1.00	5.00	0	0	170
				0	0	190
				20	0	170
				340	0	190
				40	0	170
				300	0	190
				90	0	170
7	−5.00	−1.00	5.00	0	0	170
				0	0	190
				20	0	170
				340	0	190
				35	0	170
				295	0	190
				90	0	170

**Table 2:** Isocenter, couch angle, and arc ranges of test treatment plans used for collision‐free gantry range. Eclipse isocenter coordinate is based on Varian IEC convention.

Plan no.	Eclipse isocenter (cm)			Couch angle (°)	Gantry start (°)	Gantry end (°)
	X (+: Lt)	Y (+: Post)	Z (+: Sup)			
A	0.00	0.00	−4.00	50	20	160
				80	20	160
				290	200	340
				300	200	340
B	−2.00	−2.00	−1.00	50	20	160
				60	20	160
				290	200	340
				300	200	340
C	2.00	−2.00	−1.00	60	20	160
				70	20	160
				300	200	340
				310	200	340
D	0.00	−7.50	2.00	20	20	160
				40	20	160
				320	200	340
				340	200	340
E	0.00	6.00	−4.00	20	90	270
				30	90	270
				330	90	270
				340	90	270

## 3 Results

The average deviation between the measured clearance distance and the prediction was 0.1 ± 1.0 mm for the BrainLAB ExacTrac couch system and 0.6 ± 0.7 mm for the Varian PPC system. The positive deviation means the measured distance is longer than the prediction. Only 16 out of 50 in the BrainLab Couch system and two out of 50 in the Varian Couch system showed negative deviation. The average deviation for each isocenter ranged between −0.3–0.7 mm and 0.0–0.9 mm as shown in Table 3. The minimum and maximum deviation of each system was −1 mm/+2 mm and −2 mm/+2 mm, respectively. The predicted gantry angles of minimum clearance distance were also matched in the measurement. The average deviation between the predicted collision‐free gantry angle and the measured gantry angle with a 5‐mm interval was 1.1 ± 1.1° as shown in Table 4. The positive deviation denotes that the actual gantry angle was closer to the couch top or FRPA than the predicted angle, which means an actual clearance distance at the predicted gantry angle was more than 5 mm. Only one out of 20 showed negative deviation in this measurement. Minimum and maximum deviations were −1.1° and 4.3, respectively.

**Table 3:** Deviation between the measured clearance distance and predicted distance. Positive value means a measured distance is longer than the prediction.

Plan no.	Brainlab couch + Varian cone		Varian couch + Brainlab cone	
#1	0.7 ± 1.3 mm	(0–2 mm)	0.9 ± 1.1 mm	(−1–3 mm)
#2	0.0 ± 1.3 mm	(−1–1 mm)	0.3 ± 0.8 mm	(−2–2 mm)
#3	‐0.1 ± 1.2 mm	(−1–1 mm)	0.1 ± 0.7 mm	(−1–2 mm)
#4	‐0.3 ± 0.5 mm	(0–1 mm)	0.9 ± 0.4 mm	(−1–0 mm)
#5	0.0 ± 1.3 mm	(0–1 mm)	0.0 ± 1.3 mm	(−1–0 mm)
#6	‐0.3 ± 1.0 mm	(0–1 mm)	0.9 ± 0.4 mm	(−1–1 mm)
#7	0.4 ± 1.0 mm	(0–1 mm)	0.4 ± 0.5 mm	(−1–2 mm)
Total	0.1 ± 1.0 mm	(0–2 mm)	0.6 ± 0.7 mm	(−1–3 mm)

**Table 4:** Deviation between the predicted collision‐free gantry angle and the measured gantry angle with 5‐mm interval. Positive value means the actual gantry angle was closer to the couch top or FRPA than predicted angle.

Plan no.	Gantry angle deviation	
A	0.8 ± 1.2°	(−1.1–3.3°)
B	0.7 ± 0.4°	(0.2–1.2°)
C	1.7 ± 1.5°	(0.0–4.3°)
D	0.7 ± 0.5°	(0.3–1.9°)
E	1.5 ± 0.9°	(0.5–3.2°)
Total	1.1 ± 1.1°	(−1.1–4.3°)

## 4 Discussion

Stereotactic radiosurgery planning with a stereotactic cone is always challenging due to the potential collision of the SRS cone with the patient localization device, couch, and patient body. The incidence rate of collision during the verification procedure before the plan approval was more than 50% in the SRS planning of trigeminal neuralgia based on institutional experience in BSW. In this study, the BEV‐based collision detection method implemented in the in‐house software was presented to overcome this problem. This method utilizes well‐established BEV coordinate transformation from the contour of the collisional object without complex 3D modeling, CAD technique, or external camera. Once the DICOM file, containing the 3D contours of the SRS Positioning Array and couch top, is obtained at the beginning, no additional contouring or user intervention is needed during the planning since the BrainLAB system has its own unique origin defined in its SRS coordinate system. In case the BrainLAB coordinate is not being defined in iPlan, the same manipulation is available by translating the DICOM coordinates of the outer contour about the virtual isocenter which can be found by an image registration with the couch extension template.

The average deviation between the measurement and the prediction was <1 mm for both BrainLAB ExacTrac and Varian PPC systems. Though the maximal and minimal deviation in both systems were quite similar, the average deviation was a little bit higher in Varian and more negative deviation was shown in the BrainLAB system. It seems it was caused by the inter‐observer variations at two different institutions, which could be improved by acquiring more independent measurement samples. The prediction accuracy is quite comparable with the previous studies using Kinect camera or CAD software. The accuracy inherits from the mathematical operation of BEV applied to the fixed CT coordinates of the outer contour. In camera‐based surface detection, Padilla et al showed an average discrepancy of 0.5° in detecting a collisional angle, and Cardan et al reported an average prediction accuracy of 97.3% with a raw gantry model and 91.5% with a 6 cm additional buffer.^15^, ^16^ In CAD‐based technique, Yu et al reported a maximal discrepancy of 0.95 and 2.97 cm for gantry‐to‐couch and gantry‐to‐phantom distance, respectively, and Mann et al was able to predict minimum distances within 3 cm with a safety margin of 1.5 cm.^17^, ^18^

The limitation of this study is the exclusion of potential translational, rotational errors and corrections during the patient setup. It may pose unexpected deviations on the result since the algorithm has a fixed detection margin and assumes no setup uncertainty. According to Jin et al,^25^ mean translational and rotational corrections were 2.45 mm and 1.84° in the 6D frameless BrainLAB system using x‐ray correction. If the average radius from the isocenter to the end of a setup device is presumed to be 15 cm, the resultant couch motion with 1.84° will be 4.8 mm. So, the default detection margin in the determination of the collision and collision‐free range has to be increased to 10 mm or more in clinical practice considering the sum of translation and rotational uncertainty. The detection margin itself is an adjustable value with the user's experience in the clinic though it was set to 5 mm by default. The automatic “search body” feature with a relaxed HU threshold of −650 HU was used to contour the fine structures of the FRPA. It will help to have an inherent margin in the delineation of the outer contour in addition to the detection margin. Positive deviations of 82 cases out of 100 in the clearance distance measurement and 39 cases out of 40 in the collision‐free gantry angle measurement also supported it. The maximum deviation was found to be 4.3° which amounts to a 5–6‐cm overestimation due to the HU threshold described above and a finite CT slice thickness etc. However, considering the uncertainty in the edge of the setup device or couch, it will be acceptable to have a conservative prediction as long as it is more than the safety margin.

This study aimed specifically for cone‐based SRS using the BrainLAB system, but not limited to it. From the institutional experiences, there were no collision issues in MLC‐based SRS with a dedicated head and neck couch extension. A similar approach will be applicable to the other SRS systems and treatment couches including a spine SBRT with cones. Currently, automated treatment planning and delivery of non‐coplanar SRS plans is available with the HyperArc (Varian Medical Systems, Palo Alto, CA) based on the CAD‐based collision detection algorithm.^26^ It is based on the image registration of the built‐in fiducial markers in the TPS to that of the patient CT image using the QFix^TM^ mask system. Similarly, the proposed method has a potential application to conventional external beam radiation therapy as well by combining an indexed couch position, isocenter location, patient body contour and preprocessed patient setup device into the known treatment machine geometry. Extension to proton beam therapy is also easily achievable with the measured geometry of the proton beam nozzle.

## 5 Conclusion

The proposed method was able to calculate the minimum clearance distance between the cone surface and the collisional object with <1 mm accuracy for cone‐based SRS. Collision‐free arc range also could be found accurately with a preset margin. It can be useful to provide prior information on the arrangement of collision‐free couch and arc angle to the radiation treatment planners at the time of planning.

## Conflict of interest

No conflict of interest.
